# General and Specific Aversive Modulation of Active Avoidance Require Central Amygdala

**DOI:** 10.3389/fnbeh.2022.879168

**Published:** 2022-06-20

**Authors:** Ian T. Kim, Claudia Farb, Mian Hou, Sunanda Prasad, Elyse Talley, Savannah Cook, Vincent D. Campese

**Affiliations:** ^1^Center for Neural Science, New York University, New York, NY, United States; ^2^Behavioral and Neural Sciences Graduate Program, Rutgers University-Newark, Newark, NJ, United States; ^3^Center for Molecular and Behavioral Neuroscience, Rutgers University-Newark, Newark, NJ, United States; ^4^Department of Psychology & Behavioral Sciences, University of Evansville, Evansville, IN, United States

**Keywords:** avoidance, amygdala, transfer, motivation, instrumental

## Abstract

Three studies provide evidence that the central nucleus of the amygdala, a structure with a well-established role in conditioned freezing, is also required for conditioned facilitation of instrumental avoidance in rats. First, the immediate early gene c-Fos was measured following the presentation of a previously shock-paired tone in subjects trained either on an unsignaled avoidance task or not (in addition to tone only presentations in naïve controls). Significantly elevated expression of c-Fos was found in both the avoidance trained and Pavlovian trained conditions relative to naïve controls (but with no difference between the two trained conditions). In a subsequent study, intracranial infusions of muscimol into the central amygdala significantly attenuated the facilitation of shock-avoidance by a shock-paired Pavlovian cue relative to pre-operative responding. The final study used a virogenetic approach to inhibit the central amygdala prior to testing. This treatment eliminated the transfer of motivational control over shock-avoidance by both a shock-paired Pavlovian stimulus, as well as a cue paired with a perceptually distinct aversive event (i.e., klaxon). These findings provide compelling support for a role of central amygdala in producing aversive Pavlovian-instrumental transfer.

## Introduction

Studies of aversive Pavlovian learning have produced a rich understanding of the psychological mechanisms and neural circuitry responsible for conditioned defensive reactions. Adaptive behaviors (e.g., freezing) and cardiovascular responses come under the control of a previously neutral conditioned stimulus (CS; e.g., tone) through repeated pairings with an aversive unconditioned stimulus (US; e.g., footshock; LeDoux et al., 1988). At the neural level, this depends on signals along auditory and somesthetic pathways converging in the lateral nucleus of the amygdala (LA) and engaging Hebbian plasticity that potentiates the auditory input (Rogan and LeDoux, 1996; Rosenkranz and Grace, 2002). Following training, increased CS-elicited activity in LA produces conditioned responding (CR; e.g., freezing) via connections to the central amygdala (CeA), which then projects to brainstem areas that directly stimulate the relevant behaviors (LeDoux, 2000). Indeed, much has been learned about the electrophysiological activity and various molecular processes that support this form of learning (Johansen et al., 2011; Herry and Johansen, 2014). However, aversive Pavlovian cues can serve other purposes beyond producing simple CRs, such as modulating ongoing goal-directed behavior (Bolles and Popp, 1964; Rescorla and Lolordo, 1965; Rescorla, 1968; Weisman and Litner, 1969; Overmier and Payne, 1971; Overmier and Brackbill, 1977; Patterson and Overmier, 1981). While studies of appetitive learning have elegantly examined the substrates for modulatory effects of the CS (see Cartoni et al., 2016), our understanding of how this is accomplished with aversive stimuli is very limited.

Different forms of Pavlovian-instrumental transfer (PIT; e.g., conditioned suppression and facilitation) demonstrate that an aversive Pavlovian CS can modulate ongoing instrumental behaviors (e.g., food-reinforced lever-press or shock-avoidance responding). While conditioned suppression has been studied extensively (LeDoux et al., 1990; Killcross et al., 1997; Cardinal et al., 2002; Lee et al., 2005; Elrich et al., 2012; also see Fernando et al., 2014), the neural mechanisms involved in aversive conditioned facilitation are not well understood. Using an aversive PIT task, where footshock-avoidance behavior (e.g., two-way shuttling) is enhanced by a separately trained shock-paired CS, we found that CeA is necessary for conditioned facilitation (Campese et al., 2013, 2015). However, these findings were obtained using electrolytic lesions, thus the possibility remains that nonspecific effects could have accounted for the behavioral results (but see Campese et al., 2017a). Given the role of CeA in conditioned freezing, further evidence for this opposing function (i.e., response facilitation) may prove valuable for understanding how CeA may regulate a variety of behavioral responses. Therefore, in the following studies, we sought to establish a role for CeA in aversive PIT using more selective means beyond lesions. Specifically, the immediate early gene c-Fos was quantified in CeA following PIT testing. This was compared to CeA in control subjects that had undergone Pavlovian conditioning but without shock-avoidance training. To follow this up, intracranial muscimol was used to inhibit CeA during PIT in a within-subjects design. The final study had a similar approach but used designer Kappa opioid receptors (KORD; Vardy et al., 2015; Marchant et al., 2016) controlled by the synthetic ligand salvinorin-B (Sal-B) to inhibit CeA. KORD was used because it provides a more accurate means to visualize the cells being targeted than muscimol. This study also extended the analysis to whether different forms of aversive conditioned facilitation (i.e., sensory-specific or general) depend on CeA. Appetitive procedures that isolate sensory-specific and general PIT have led to important discoveries about how distinct neural pathways process different elements of motivation (Cartoni et al., 2016). Identifying the degree of similarity in how the aversive analogs of these motivational substrates are generated at a neural level may prove similarly enlightening. The findings from these studies provide strong evidence that CeA is necessary for the modulation of avoidance behavior through both general and specific motivational mechanisms.

## Methods

### Subjects

Fifty-four male Sprague-Dawley rats were used as subjects for the studies reported below. Rats were obtained from Hilltop Lab Animals (Scottsdale, PA) and weighed approximately 300 g at the start of behavioral training. Subjects were housed individually in standard paper bedding lined Plexiglass cages in a colony running a 12:12 h light-dark schedule with access to free food and water. Animal care and housing met the current standards of the International Association for Assessment and Accreditation of Laboratory Animal Care (AAALAC). All procedures reported herein were pre-approved by the New York University Animal Welfare Committee.

### Apparatus

Subjects were trained in chambers manufactured by Coulbourn Instruments (Whitehall, PA) running Graphic State 3 (Actimetrics) software to control the sessions and measure responding. All chambers included stainless steel grid floors for shock delivery as well as an 8 ohm speaker for the presentation of the 5 kHz tone and white noise stimuli. Coulbourn precision animal shockers (model no. H13-15) and programmable audio generators (model no. A12-33) were used for stimulus delivery. For Pavlovian training, standard chambers (26 × 28 × 20 cm, length × width × height; model no. H10-11R-TC), each equipped with a klaxon horn (model no. 330; 114dB) made by Wolo (Deer Park, NY) were used. Footshock avoidance took place in two-compartment shuttlebox chambers divided by a panel with a threshold cutaway for passage (50.8 × 25.4 × 30.5 cm, length × width × height; model no. H10-11R-SC). Avoidance chambers were equipped with infrared emitter-detector arrays to capture responses automatically. All chambers were individually housed in light and sound attenuation cubicles (model no. H10-24C). Pavlovian training and transfer test sessions were recorded using a digital video recorder (model no. DVR814) purchased from CCTV Imports (Madisonville, LA) for quantification of freezing.

### Procedure

#### Behavioral Training

The studies reported here involved a combination of aversive Pavlovian conditioning, active avoidance, and transfer testing where the effect of the Pavlovian stimuli on avoidance rates were evaluated. Because there were minor modifications to these procedures for reasons specific to each study, a general description of the tasks will be provided with details offered for the changes required in each study.

#### Pavlovian Threat Conditioning

Subjects received Pavlovian threat conditioning (PTC) in standard chambers (context A). PTC for a given cue was conducted in a single session where a 30 s 5 kHz tone co-terminated with a 1 s 0.6 mA footshock US. There were three trials following a 5-min baseline separated by a 3-min intertrial interval (ITI) and sessions were 15-min in duration.

##### Fos Study

Subjects in both the PAV and PIT groups of this study received tone-shock training as described above (controls received no training) on the first day of the experiment. At the end of the session, subjects were removed from the chambers and returned to their home cages. The USAA phase of the study began 24 h later.

##### Muscimol Study

All subjects in this study received tone-shock training as described above and were returned to the colony for the rest of the day following training. The USAA phase began the following day.

##### KORD Study

Subjects in this study received two PTC training sessions over the first two days of training (one each day). Over these sessions, half of the subjects received tone-shock and noise-klaxon pairings, while these relationships were reversed for the other half. The order of these sessions was counterbalanced during training as were the specific stimuli. In other words, half of each counterbalanced subgroup had tone trained on day 1 and noise on day 2, while this was reversed in the other half of the subjects. For klaxon training, all other parameters were maintained, with the difference being the replacement of the footshock with a 5 s klaxon delivery.

#### Unsignaled Sidman Active Avoidance

Subjects underwent Unsignaled Sidman Active Avoidance (USAA) training in two-way shuttle chambers (context B) over the next 15 days of the study. During these sessions, 0.5 s footshocks (0.6 mA) were programmed for delivery every 5 s (Shock-Shock or S-S interval). However, each shuttling response delayed the delivery of the next shock by 30 s (Response-Shock or R-S) interval. Thus, rats could prevent the delivery of all shocks by shuttling at least once every 30 s. Avoidance responses were defined as shuttles during the R-S interval, while shuttles made during the S-S interval were defined as escape responses. Each shuttle response was accompanied by a brief 0.3-s blinking houselight to provide feedback to the subject (Sidman, 1953a, b). Daily sessions were 25-min in duration and concluded with the houselight turning off (see Lázaro-Muñoz et al., 2010).

##### Fos Study

Subjects in the PIT group received USAA training as described above. Subjects in the PAV group were not trained, but were instead, only placed in the avoidance chambers and received no shocks during this phase. For these subjects, shuttling still caused the house lights to blink, but this was not associated with safety. Box control subjects remained in their home cages during this phase.

##### Muscimol Study

All subjects in this study received USAA as described above.

##### KORD Study

All subjects in this study also received USAA as described above. For subjects given USAA training in all studies reported here, only those that made 20 or more avoidance responses in consecutive sessions within the first 10 days of training were tested for transfer. If a subject did not meet this requirement, they were excluded from further training and all analyses. In total, eight rats were excluded from analyses due to poor performance.

#### Pavlovian-Instrumental Transfer

To test for aversive transfer (i.e., PIT), shuttling responses during CS-free and CS periods were compared during extinction sessions (no shocks) in context B (Campese et al., 2013). Each test session began with a 15-min baseline period, after which, the CS presentation occurred once the shuttling rate dropped below two responses per minute for two full minutes. The CS then remained on until 10 shuttle responses were made, at which point, the houselight turned off and the session ended. Note that response-produced feedback stimuli were presented during tests as in training. For PIT tests, responses per minute data were used for both presentation and analysis.

##### Fos Study

In this study, there was a single PIT test using the procedure described above. While previous studies (and those below) involve multiple test sessions to evaluate transfer, a single test was used here to capture the first instance of transfer for the purpose of c-Fos measurements. It may be the case that as transfer tests accumulate, there could be learning effects that emerge, especially given that responding terminates test sessions. Given this possibility, differences in Fos expression could be seen as a function of whether one or multiple tests are used. The single test was followed by perfusion and brain removal for c-Fos analysis. Because subjects in the Fos study only had one test session, total time freezing to the CS during this test was measured. For all subjects, the tone remained on until 10 responses were made.

##### Muscimol Study

In this study, subjects received two PIT tests on consecutive days following USAA, but prior to cannula implantation surgery. Following a 1-week recovery from surgery, subjects were given additional testing with intracranial treatments. Two tests on consecutive days were conducted following vehicle or muscimol infusions. Then, 1 week later, another two tests were conducted on consecutive days using the other infusion treatment. The postoperative test rounds were separated by one-week to encourage response recovery. Two test videos from this study were lost due to hard drive errors. The rest were scored for freezing by a trained blind rater. Because multiple tests were conducted in this study, only the 1st min of the CS was scored for these test sessions.

##### KORD Study

For this phase, subjects underwent a total of eight individual transfer tests, arranged into four blocks with two tests in each block. The first four tests (i.e., blocks 1 and 2) were conducted with tone, and the last four tests (i.e., blocks 3 and 4) with the white noise CS. For all subjects, tone was tested on the first two consecutive days of the test phase. This was followed by two additional tone test sessions 5 days later. The interpolated time between tests was meant to encourage response recovery. Noise testing began the day after the final tone test and was done in the same way (i.e., with 4 days off between tests involving different drug assignments). Subjects received a given IP treatment (i.e., Veh or sal-B) on consecutive days and were tested with the same CS on both days. This was counterbalanced for drug assignment over the test phase so that each subject produced a PIT score (comprised of a two-test average) for tone and noise under both vehicle and Sal-B treatments. Drug assignments were arranged to account for block order, so that if for example, a subject had vehicle treatment before tests 1 and 2, and sal-B for tests 3 and 4, this was reversed for the noise tests, resulting in sal-B treatment for tests 5 and 6, with vehicle prior to tests 7 and 8. It should be noted that this arrangement resulted in half of the subjects being tested for CS-shock while the other half were tested for transfer to CS-klaxon during each session. This scheme was chosen because previous findings suggest noise is more effective at driving transfer. Given the many sessions needed to produce within-subjects comparisons for KORD status, stacking noise sessions in the final blocks was aimed at avoiding a floor effect. A single video from this test phase was lost due to a recording error. Freezing was evaluated by a blind rater measuring the first min of each trial during each test.

### Surgery

#### Cannulations and Intracranial Treatments

Following baseline PIT testing for subjects treated with muscimol, rats were anesthetized with a mixture of ketamine (100 mg/kg: Vedco) and xylazine (10 mg/kg) via intraperitoneal (IP) injection (0.1% bodyweight). Subjects were placed in a Kopf (David Kopf Instruments; Tujunga, CA) stereotaxic instrument, and an incision was made over the midsagittal line to reveal bregma and lambda on the surface of the skull. Stainless steel (22 gauge) guide cannula (Plastics One, Roanake VA) were fixed in place with jeweler’s screws and dental cement 1.5 mm above CeA at -2.5 mm posterior, 4 mm lateral to the midline, and 6 mm ventral from the surface of the skull. Subjects recovered in the home cage for one week following surgery and then underwent further PIT testing. Prior to testing, muscimol (0.3 μl of 1 ng/nl solution) or deionized water was infused through (28 gauge) injectors extending 1.5 mm beyond the guides bilaterally at a rate of 0.15 μl/min with subjects connected to the infusion lines for an additional min for dispersal. This was accomplished using 10 μl Hamilton syringes (Model 701N) controlled by a Harvard Apparatus pump (PHD 22/2000) via polyethylene tubing connected to injectors extending 1.5 mm beyond the tip of their guide cannula. Subjects were tested 15–20 min after treatment.

#### Viral Injections

Prior to behavioral training subjects were anesthetized and prepared in the stereotaxic apparatus as described above. Through a 1 μl Hamilton Neuros syringe, 0.7 μl of AAV9 CamKII containing instructions for Gi-coupled modified kappa opioid receptor (KORD; Vardy et al., 2015; Marchant et al., 2016) was injected bilaterally into CeA over 5 min and allowed to spread for an additional 5 min before removing the needle. The incision was sutured and subjects were given 2 weeks to recover in the home cage prior to undergoing behavioral training. To engage KORD receptor-based neural inhibition, 20–30 min prior to PIT testing, subjects received a 0.1% bodyweight injection of 5 mg/kg IP salvinorin-B (Sal-B) or vehicle for the control treatment. Sal-B was purchased from Applepharms (Asheville, NC) and dissolved in 7% DMSO (Sigma Aldrich, St. Louis MO), then added to a 50–50 deionized water-polyethylene glycol (Sigma Aldrich, St. Louis, MO) mix at 40°C.

#### c-Fos Immunocytochemistry

**Figure 1:**
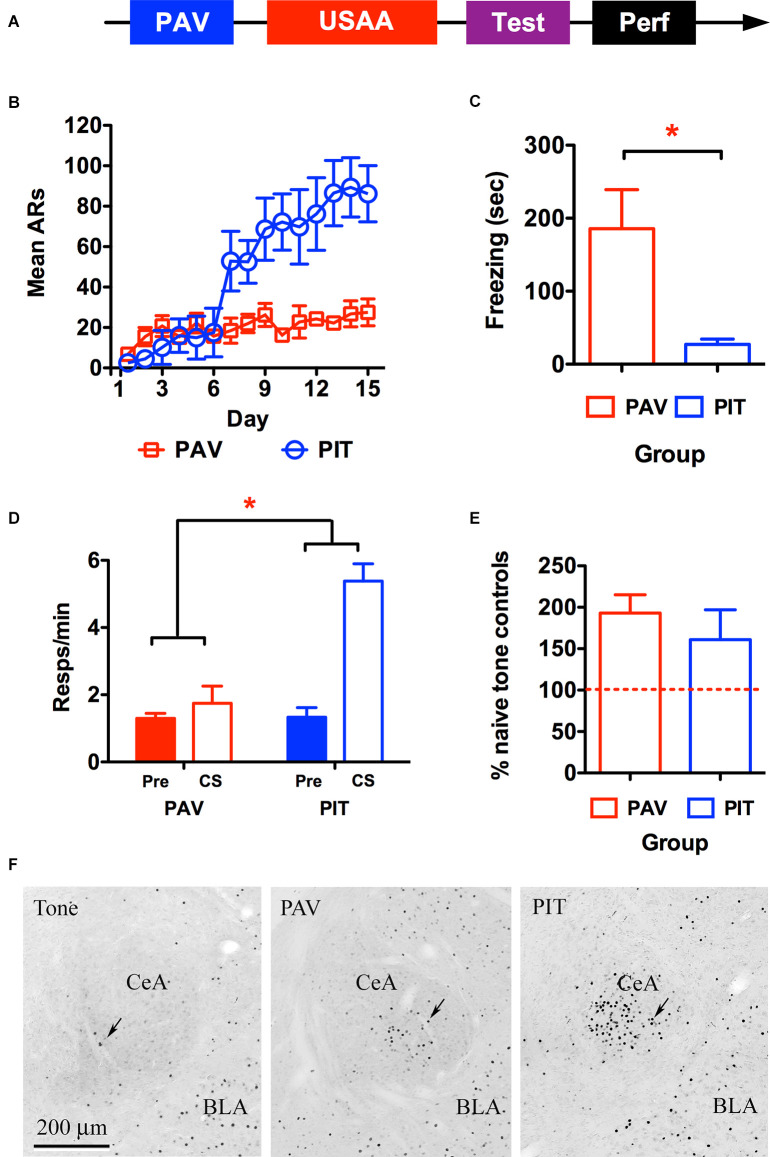
Timeline for the behavioral procedure used to quantify c-Fos is shown in panel **(A)**. **(B)** Shuttling behavior depicts acquisition of avoidance responding during the Sidman (USAA) training phase for the avoidance-trained (PIT) and Pavlovian-only (PAV) control subjects. Freezing behavior **(C)** and shuttle responding **(D)** during the transfer test (Pre CS and CS) are presented for each group as well as average Fos counts per group expressed as a percentage of baseline Fos seen in the naïve control subjects exposed only to tone prior to perfusion **(E)**. Panel **(F)** shows representative images of amygdalae in Tone, PAV, and PIT groups from left to right. ARs, avoidance responses. Asterisks denote significance at the 0.05 alpha level.

Ninety minutes after the end of behavioral testing, animals were anesthetized with the ketamine and xylazine mixture and transcardially perfused with approximately 30 ml of phosphate-buffered saline (PBS; 0.01 M phosphate buffer, pH 7.4), followed by approximately 300 ml of 4% paraformaldehyde (PFA). The brains were removed from the skull, post-fixed in PFA, and were cut into 50 μm coronal sections on a Vibratome (Leica, Germany). Tissue sections containing the CeA were collected in PBS with 0.05% sodium azide and stored at 4°C. Tissue sections were incubated for 30 min in 1% bovine serum albumin (BSA; Sigma Aldrich, St. Louis, MO) made in PBS to block nonspecific binding and then incubated overnight in polyclonal rabbit anti-c-Fos primary antiserum (Calbiochem; 1:10,000). Following the incubation, sections were rinsed, incubated for 30 min in biotinlyated goat anti-rabbit IgG (1:200; Vector Laboratories, Burlingame, CA), rinsed, and incubated for 30 min in the avidin–biotin–horseradish peroxidase complex (ABC; VECTASTAIN Elite Kit, Vector). Staining was visualized using the chromogen Very Intense Purple (VIP; Vector Laboratories). Primary and secondary antibody incubations were made in 1% BSA/0.05% sodium azide/PBS and the primary incubation contained 0.2% Triton-X. Sections were mounted on gelatinized slides, dehydrated briefly in 100% ethanol, defatted in xylene, and coverslipped with Permount (Fisher Scientific, Hampton, NH). High resolution, digital images were acquired at 10X using the VS120 Virtual Slide Microscope (Olympus) (see Figure 1F for examples). The CeA was defined according to the Paxinos and Watson rat brain atlas (Paxinos and Watson, 2005) and sampled from the most posterior to the most anterior levels (Bregma −3.36 to −1.44). For each section, the surface area of the CeA was measured using FIJI (Image J software; National Institute of Health, Bethesda, MD) and an experimenter blind to the conditions manually counted the number of labeled Fos using the Image J “qwertyujk90-= cell counter” plug-in. For each experimental condition, the total number of Fos-positive cells were counted bilaterally and expressed as the number of Fos-positive cells per unit area.

#### Perfusions and Immunocytochemistry

Subjects were deeply anesthetized with the mixture of the ketamine/xylazine mixture and transcardially perfused. For cannulated subjects treated with muscimol prior to testing, 0.01 M PBS was followed by 10% formalin during perfusions (Fischer Scientific). Brains were removed from the skull and postfixed in 10% formalin. Fluorescent muscimol (Sigma Aldrich, St. Louis, MO) infusions were made 10 min prior to perfusion using the same parameters described earlier. 50 μm coronal sections were made on a Vibratome and stained for thionin to identify injection sites or left untreated and coverslipped on a slide to visualize muscimol spread using fluorescence. For KORD expressing rats, subjects were perfused with 0.01 M PBS and then 4% PFA and brains post-fixed in PFA. Brains were cut into 50 μm coronal sections made on a Leica Vibratome and stored in PBS with 0.05% sodium azide and kept at 4°C until processing. Expression of KORDs was visualized using a rabbit anti-GFP antibody (1:2K; #A11122; Life Technologies), biotinylated goat anti-rabbit IgG (1:200; Vector Labs), avidin-biotin horseradish complex (Elite ABC Kit; Vector Labs) and Very Intense Purple (VIP Kit; Vector Labs). Sections were mounted, coverslipped, and viewed using a high-resolution digital camera microscope system.

## Results

### Expression of Immediate Early Genes in Central Amygdala During Transfer of Motivational Control

To test for CeA activity in aversive transfer, the immediate early gene c-Fos was measured in relation to the presentation of a previously shock-paired tone on active avoidance behavior (i.e., two-way shuttling). Subjects underwent the sequence of training depicted in Figure 1A below involving aversive auditory Pavlovian conditioning in the first phase, where a tone CS was paired with a footshock US. This was followed by USAA training, where shuttle responding was negatively correlated with footshock. Finally, during the transfer test, the CS was presented during USAA performance (in the absence of shock) and the effect of the cue on shuttling rates was quantified. Following this session, subjects were perfused and c-Fos expression in CeA was measured in relation to this event. Based on our findings that CeA lesions impaired PIT (Campese et al., 2014, 2015) we anticipated that PIT testing would result in c-Fos expression in CeA. However, because CeA is well known for its role in freezing CRs, we evaluated this relative to control subjects that had undergone Pavlovian conditioning but did not have USAA training. This was done to determine whether USAA training quantitatively changes CS-processing-related CeA activity during PIT. Below, these rats are referred to as PAV subjects, whereas those that received avoidance are referred to as PIT subjects.

#### Avoidance Training

Two subjects were excluded from the PIT group due to poor USAA performance and one due to inadequate perfusion. The final sample size was *n* = 4 for the PAV group and *n* = 5 for the PIT group. Shuttling data from the USAA phase are presented in Figure 1B below. Avoidance responding increased over this phase but did so significantly more for subjects given USAA training compared to non-shocked USAA exposure. This was confirmed by a mixed repeated measures analysis of variance (rmANOVA) including *Day* (1–15) as a within-subjects factor and *Group* (PAV vs. PIT) as a between-subjects factor (*F*_Day__(14,98)_ = 10.85, *p* < 0.001; *F*_Group (1,7)_ = 4.1, *p* = 0.08; *F*_Interaction__(14,98)_ = 7.08, *p* < 0.001).

#### Transfer Test

Mean freezing during the CS is presented in Figure 1C below for the PAV and PIT groups. Significantly more freezing was seen in subjects that did not receive USAA training (*t*_2-tailed__(7)_ = 3.32, *p* = 0.01). Rather than freeze to the CS, USAA trained subjects showed enhanced avoidance responding instead (see Figure 1D). This was confirmed by a mixed rmANOVA including *Interval* (Pre vs. CS) as a within-subjects factor and *Group* (PAV vs. PIT) as a between-subjects factor (*F*_Interval__(1,7)_ = 24.5, *p* < 0.01; *F*_Group (1,7)_ = 12.05, *p* = 0.01; *F*_Interaction__(1,7)_ = 18.81, *p* < 0.01). Follow-up Bonferroni corrected comparisons localized this effect to higher shuttle responding during the CS for PIT subjects compared to responding in all other intervals for both groups [Pre CS; *M*_Pav_ = 1.3, 95% CI(0.6 2.0), *M*_PIT_ = 1.24, 95% CI(0.6 3.2); CS; *M*_Pav_ = 1.75, 95% CI(0.3 1.87), *M*_PIT_ = 5.26, 95% CI(3.97 6.56)]. It should be noted that USAA training and the subsequent transfer effect in the PIT group enabled these subjects to end their transfer test earlier than the two control groups, by executing 10 responses more quickly. Means for CS duration during the test are 8.1, 8.6, and 2.0 min in the Pav, Tone (naïve control), and PIT groups respectively.

#### c-Fos Analysis

Preliminary analyses showed no effect of the Hemisphere, so data were collapsed across this factor. These data are presented in Figure 1E as percent labeling relative to the naïve control group (i.e., Tone) (*n* = 4) exposed to the tone CS in the avoidance arena during the test. There were no differences in c-Fos labeling in CeA between the PAV and PIT groups, *t*_(7)_ = 0.71, *p* = 0.50.

### Intracranial Muscimol in CeA Eliminates Aversive PIT

**Figure 2:**
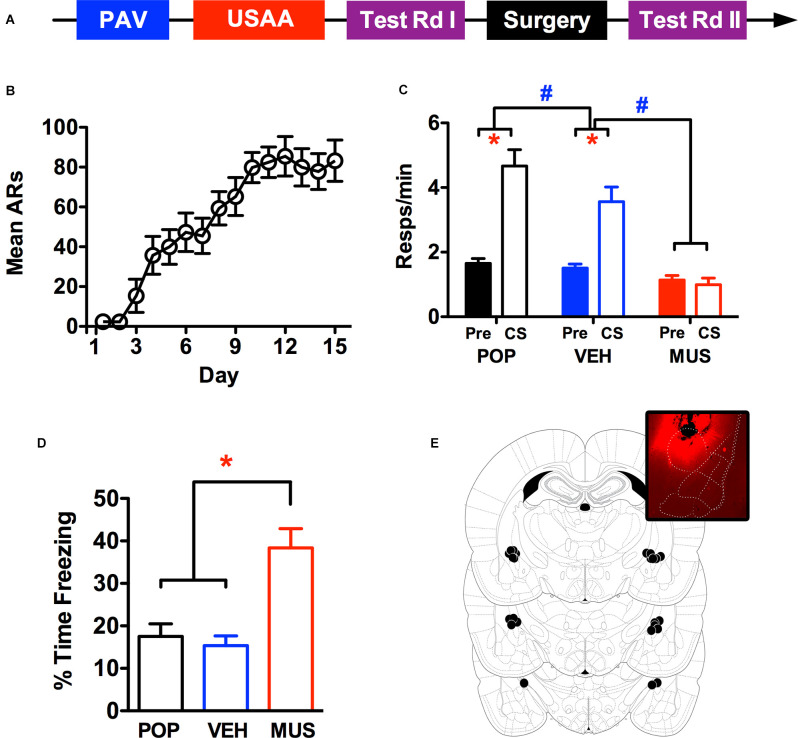
Panel **(A)** shows the timeline for the behavioral procedure used to test for the effects of CeA inactivation via intracranial muscimol infusions. Panel **(B)** shows the acquisition of avoidance over the USAA phase, while panel **(C)** shows transfer test responding (Pre CS and CS) in the preoperative (POP) as well as postoperative test sessions preceded by treatment with vehicle (VEH) and muscimol (MUS). Percent time freezing during the CS in the transfer test phase (POP, VEH, and MUS) are presented in panel **(D)** and injection sites in the amygdala are summarized in panel **(E)**, the inset shows representative spread using fluorescent muscimol prior to perfusion (Figure adapted from Paxinos and Watson (2005), with permission from Elsevier). ARs, avoidance responses. Asterisks indicate significant effects at alpha = 0.05. Pound signs reflect significant effects with a 5% alpha, but for comparisons of overall responses across the tests showing postoperative muscimol rates lower than all other tests.

Previous studies with electrolytic lesions have found that CeA is important for the aversive transfer effect (Campese et al., 2014, 2015). To provide converging evidence of this, in the current experiment we used reversible intracranial inhibition by the GABA_A_ agonist muscimol to temporarily disrupt the activity of CeA neurons prior to PIT testing. Subjects were trained and given baseline PIT testing prior to cannula implantation and then tested again after recovery following infusion of muscimol or vehicle into CeA (see Figure 2A). Based on our previous work, we expected that muscimol treatment would impair the facilitative effect of an aversive CS on avoidance behavior.

#### Avoidance Training

Acquisition of footshock-avoidance responding proceeded normally over this phase. Three subjects were eliminated from the analyses due to poor USAA performance and 13 remaining subjects were included in the data reported for this study. Mean avoidance responding over training is summarized in Figure 2B. Over training, responding steadily increased, an impression confirmed by a rmANOVA including *Day* as a within-subjects factor, *F*_(14,168)_ = 18.31, *p* < 0.001.

#### Transfer Testing

Shuttling data from the test phase are presented in Figure 2C for the preoperative (POP) as well as the postoperative vehicle (VEH) and muscimol (MUS) test waves. Preliminary analyses found no significant effects involving test order (*F*_Test__(1,12)_ = 0.45,*p* = 0.52; *F*_Test × Interval__(1,12)_ = 0.001, *p* = 0.97) so data were collapsed across this factor and are presented as combined averages over the two tests in each wave. A rmANOVA including the within-subjects factors of *Wave* (POP, VEH, MUS) and *Interval* (Pre vs. CS) confirmed the impression that while PIT was normal following vehicle infusion, it was abolished by CeA muscimol infusions (*F*_Wave__(2,24)_ = 38.8, *p* < 0.001; *F*_Interval (1,12)_ = 23, *p* < 0.001; *F*_Interaction__(2,24)_ = 17.9, *p* < 0.001). Follow-up Bonferroni corrected comparisons showed that while overall responding was comparable between preoperative and vehicle tests (*p* = 0.10), responding after muscimol treatment was significantly lower (*p* < 0.001). Inspection of the interaction effect revealed that responding during the CS was slightly reduced following surgery but much more so following muscimol than vehicle infusions [CS—*M*_POP_ = 4.67, 95% CI(3.57 5.77), *M*_VEH_ = 3.56, 95% CI(2.57 4.55), *M*_MUS_ = 0.99, 95% CI(0.55 1.44)]. While responding during the CS was significantly higher than pre CS responding for preoperative and vehicle testing, this was not the case for muscimol testing [Pre CS—*M*_POP_ = 0.15, 95% CI(1.33 1.98), *M*_VEH_ = 1.51, 95% CI(1.23 1.78), *M*_MUS_ = 1.14, 95% CI(0.84 1.44)]. This analysis confirms that muscimol inhibition of CeA eliminates PIT.

Percent time freezing over PIT testing during the first min of the CS is presented in Figure 2D. Freezing analysis was limited to the first min of CS testing because subjects received multiple tests with variable CS durations dependent on response rate. Two 30 s bins were analyzed because the CS duration during training was 30 s. However, since freezing was comparable in both intervals, they were ultimately collapsed across this factor for presentation. While freezing was generally low throughout testing, more freezing was seen following muscimol treatment independent of the interval. This impression was confirmed by a rmANOVA including *Wave* (POP, VEH, MUS) and *Interval* (1st 30 s vs. 2nd 30 s of CS) as within-subjects factors (*F*_Wave__(2,22)_ = 16.51, *p* < 0.001; *F*_Interval (1,11)_ = 0.34, *p* = 0.57; *F*_Interaction__(2,22)_ = 1.47, *p* = 0.25). Follow-up Bonferroni corrected comparisons found that overall freezing was higher following muscimol treatment than vehicle treatment (*p* = 0.004) and prior to surgery (*p* = 0.001). Cannula placement and an example of infusion spread with fluorescence are presented in Figure 2E.

### Chemogenetic Inhibition of CeA Impairs General and Sensory-Specific Aversive PIT

**Figure 3:**
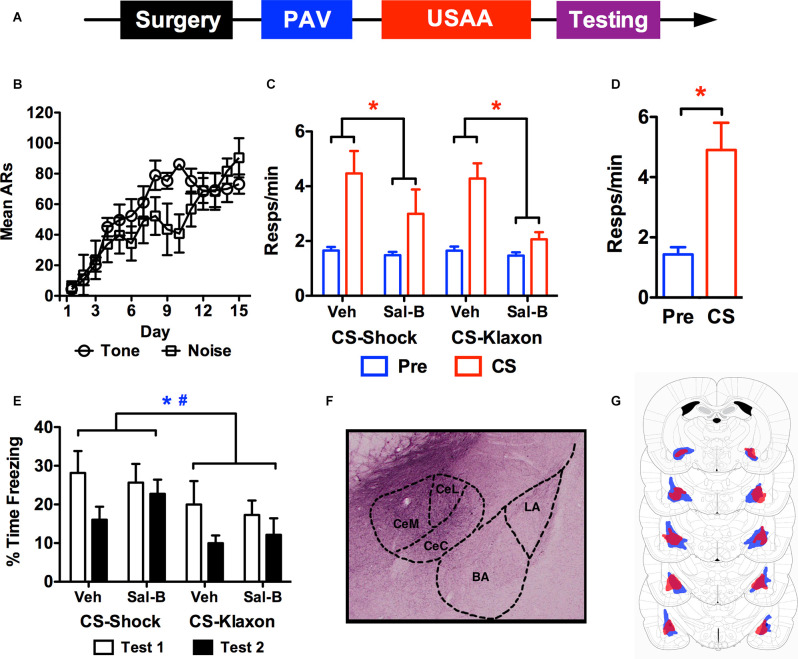
The timeline used for studying the effects of chemogenetic inactivation of the central amygdala on aversive PIT is provided in panel **(A)**. Shock avoidance acquisition is presented in panel **(B)** as a function of whether the shock was trained with tone or noise during the Pavlovian phase. Panel **(C)** shows shuttling during the transfer test phase for each CS under each treatment condition. Panel **(D)** shows responses in control subjects treated with Sal-B in the absence of KORD expression. Freezing during the transfer test for KORD expressing subjects is presented in panel **(E)**. While a representative photo of KORD expression in CeA is presented in panel **(F)**, the minimum (red) and maximum (blue) extent of viral expression for KORD is seen in panel **(G)** (Figure adapted from Paxinos and Watson (2005), with permission from Elsevier). ARs, avoidance responses. Asterisks and pound signs indicate significant effects at alpha = 0.05 for *vehicle vs. Sal-B* comparisons and *CS-shock vs. CS-klaxon* comparisons respectively.

To extend the analysis of the role of CeA in PIT, different forms of aversive transfer were studied by using distinct aversive outcomes during Pavlovian conditioning. In studies of appetitive motivation, general and sensory-specific PIT have been found to depend on parallel pathways in the amygdala and striatum (Corbit and Balleine, 2005). However, very little is known about the neural basis of these different forms of *aversive* motivation. We have recently demonstrated that a CS paired with a klaxon can similarly augment footshock avoidance (Campese et al., 2017b). This effect is more dependent on general motivation than sensory-specific features of the shock outcome. In the current study, subjects had two different CSs (tone or white noise) paired with two distinct USs (footshock or klaxon) in separate PTC training sessions. Following USAA, both the shock-paired and klaxon-paired CSs were individually tested for their ability to augment footshock avoidance. To test subjects multiple times with the two stimuli over this phase, a chemogenetic approach was used to inhibit CeA. This was chosen over muscimol because repeated infusions can damage the area of interest and limit drug dispersal. KORD was surgically infused into CeA prior to any behavioral sessions, and after a 2-week recovery period, subjects underwent the training sequence depicted in Figure 3A. Prior to PIT testing subjects were systemically treated with vehicle and the designer ligand salvinorin-B using a fully counterbalanced within-subjects approach to examine the role of CeA in the different forms of PIT.

#### Avoidance Training

Data from the USAA phase are presented in Figure 3B as a function of the CS that predicted shock during Pavlovian training. Three subjects were excluded from the analysis due to poor USAA performance, leaving 13 subjects in the final sample (tone-shock *n* = 6, noise-shock *n* = 7). Overall acquisition proceeded successfully and similarly for both counterbalanced subsets. This was confirmed by a mixed rmANOVA with *Day* (1–15) as a between-subjects factor and *Group* (Tone vs. Noise) a between-subjects factor (*F*_Day__(14,154)_ = 11.9, *p* < 0.001; *F*_Group (1,11)_ = 1.54, *p* = 0.24; *F*_Interaction__(14,154)_ = 1.58, *p* = 0.09).

#### Transfer Testing

Mean shuttling data from the PIT testing phase are presented in Figure 3C following vehicle and Sal-B treatment for all subjects. Overall, chemogenetic inhibition of CeA severely impaired the aversive transfer effect elicited by both the shock-paired and klaxon-paired stimuli. This was confirmed by a rmANOVA including *Treatment* (vehicle vs. Sal-B), *Stimulus* (CS-shock vs. CS-klaxon), *Interval* (Pre vs. CS) as within-subjects factors (*F*_Treatment__(1,12)_ = 25.34, *p* < 0.001; *F*_Stimulus (1,12)_ = 0.5, *p* = 0.50; *F*_Interval__(1,12)_ = 18.4, *p* = 0.001). While the *Treatment* × *Interval* interaction was significant, *F*_(1,12)_ = 18.28, *p* = 0.001, no other significant effects were found (*F*_Treatment × Stimulus__(1,12)_ = 1.71, *p* = 0.22; *F*_Stimulus × Interval (1,12)_ = 0.3, *p* = 0.59; *F*_Treatment × Stimulus × Interval (1,12)_ = 0.76, *p* = 0.40). Bonferroni corrected comparisons found that there was more responding overall following vehicle than Sal-B treatment (*p* < 0.001) and more CS than pre-CS responding (*p* = 0.001). Furthermore, examination of the Treatment × Interval interaction showed that while baseline responding was comparable (*M*_Vehicle_ = 1.65, 95% CI(1.47 1.83), *M*_Sal-B_ = 1.48, 95% CI(1.26 1.69)], Sal-B treatment significantly attenuated responding during the CS compared to control treatment with the vehicle (*M*_Vehicle_ = 4.38, 95% CI(3.32 5.44), *M*_Sal-B_ = 2.53, 95% CI(1.5 3.56)]. In this analysis, responding was collapsed over test order since preliminary analyses found no significant effects involving this factor alone or with the *Treatment* (vehicle vs. Sal-B) factor (*F*_Test__(1,12)_ = 3.55, *p* = 0.084; *F*_Test × Treatment (1,12)_ = 2.87, *p* = 0.12). A significant Test × Interval interaction was found (*F*_Test × Interval__(1,12)_ = 6.491, *p* = 0.026), but this effect reflected differences in responding to the different CSs (tone vs. noise) and did not interact with treatment *F*_CS × Test × Treatment (1,12)_ = 0.22, *p* = 0.89. Responding was also collapsed across stimulus identity and is presented as a function of the predicted outcome (i.e., CS-klaxon, or CS-shock). In agreement with previous findings (Campese et al., 2017b), preliminary analyses found that there was generally more responding elicited by the noise than the tone (*F*_CS (1,12)_ = 10.23, *p* < 0.01; *F*_CS × Interval__(1,12)_ = 14.34, *p* < 0.01), but this did not interact with treatment effects or the signaled outcome (*F*_CS × Interval × Treatment (1,12)_ = 1.73, *p* = 0.21). To test whether non-specific effects of Sal-B caused this behavioral effect, a group of six non-operated controls were tested for PIT following Sal-B treatment (Figure 3D). Performance was normal in these subjects (*t*_(5)_ = 3.69, *p* = 0.01), suggesting that effect of the Sal-B ligand on KORDs in CeA specifically (i.e., neural inhibition) was responsible for the impaired transfer.

Freezing data from the test phase are presented in Figure 3E and extent of viral spread in Figure 3G (a representative histological image is presented in Figure 3F). While preliminary analysis of the transfer data showed no effect of test order, freezing data did show this effect. Therefore, the data are presented for tests 1 and 2 for each stimulus and treatment. While freezing was quantified the same way as described above, the data were again collapsed over the two 30 s intervals since freezing did not change on this basis. Overall, freezing was higher for CS-shock than CS-klaxon, and for test 1 than test 2. These impressions were confirmed by a rmANOVA including *Stimulus* (CS-shock vs. CS-klaxon), *Test* (1 vs. 2), *Interval* (1st 30 s vs. 2nd 30 s), and *Treatment* (vehicle vs. Sal-B) as within-subjects factors (*F*_Stimulus__(1,11)_ = 4.95, *p* = 0.048; *F*_Test (1,11)_ = 8.2, *p* = 0.015). No other significant main effects (*F*_Treatment__(1,11)_ = 0.19, *p* = 0.67; *F*_Interval (1,11)_ = 1.06, *p* = 0.33) or interactions were found (*F*_Stimulus × Treatment (1,11)_ = 0.11, *p* = 0.75; *F*_Stimulus × Test (1,11)_ = 0.001, *p* = 0.98; *F*_Treatment × Test (1,11)_ = 3.07, *p* = 0.11; *F*_Stimulus × Interval (1,11)_ = 0.18, *p* = 0.68; *F*_Treatment × Interval (1,11)_ = 0.53, *p* = 0.48; *F*_Interval × Test (1,11)_ = 0.74, *p* = 0.41; *F*_Stimulus × Treatment × Test (1,11)_ = 0.66, *p* = 0.44; *F*_Stimulus × Treatment × Interval (1,11)_ = 1.38, *p* = 0.27; *F*_Stimulus × Interval × Test (1,11)_ = 0.48, *p* = 0.50; *F*_Interval × Treatment × Test (1,11)_ = 0.04, *p* = 0.85; *F*_Stimulus × Treatment × Interval × Test (1,11)_ = 0.06, *p* = 0.80). Thus, while freezing reduced over the test phase, it did so comparably for both stimuli (CS-shock vs. CS-klaxon) and without any effect of Treatment (vehicle vs. Sal-B).

## Discussion

Together, the studies above extend previous lesion findings (Campese et al., 2014, 2015) and provide compelling evidence that CeA is important for the modulation of shock-avoidance behavior by an aversive CS. The findings from the study quantifying c-Fos clearly demonstrate the behavioral effects of USAA on subsequent aversive CS-elicited responding. Avoidance training reduced conditioned freezing to the CS and augmented footshock-avoidance shuttle responding compared to subjects that had only undergone Pavlovian conditioning; these subjects showed standard freezing CRs without any modulation of shuttling by the CS. While previous studies have shown that Pavlovian learning is required for the aversive transfer effect (Campese et al., 2013), the current findings show that successfully acquiring avoidance is also needed to produce the transfer effect.

While CeA is well known for its role in freezing CRs, there was no difference in c-Fos labeling between high-freezing/low-shuttling PAV subjects and low-freezing/high-shuttling PIT subjects. Thus, changes to CS-processing in CeA may be crucial for producing active rather than reactive CS-elicited behavior but they may not simply translate to changes in overall CeA activity. In a related finding, we have recently reported that neuromodulatory regulation of CeA by noradrenaline from the brainstem determines whether aversive PIT or conditioned freezing is expressed when the CS is tested (Campese et al., 2017a). If norepinephrine levels are increased, subjects revert to freezing CRs and PIT is reduced, suggesting that changes to CS-processing in this region may underlie the effect.

Sample sizes, while not large, were nevertheless comparable to other studies using c-Fos to evaluate the neural circuitry underlying active avoidance and provide an adequate basis for this purpose (Martinez et al., 2013). However, a more thorough analysis with larger sample sizes may reveal patterns within CeA insofar as to how responses may be distributed among the complex disinhibitory microcircuitry of this region as a function of avoidance training (Fadok et al., 2018). Whether the maintained activity in CeA drives responding after avoidance training cannot be assessed using the Fos approach. Therefore, the following study used muscimol to inactivate CeA prior to testing to determine whether this structure is necessary for aversive PIT, as previous lesion findings suggest (Campese et al., 2014, 2015).

These findings showed that aversive PIT was intact following CeA cannulations and not impaired by the surgical procedure itself. Responding to the CS was slightly attenuated 1 week following surgery, but this was likely due to extinction as the modulatory effect relative to the baseline period was preserved. This was not true following muscimol treatment, after which, the effect of the CS on shuttling was eliminated entirely. CeA is well-known for its role in freezing CRs, and it was surprising that muscimol treatment elevated freezing compared to preoperative and vehicle testing. Infusions were mostly restricted to CeA and did not spread significantly to LA or BA, providing further evidence that CeA is necessary for aversive PIT. However, it is also possible that elevations in freezing (and reductions in avoidance) were due to motor impairments and other non-specific effects of muscimol treatment. This was addressed by using KORD in the subsequent study, which also extended the examination of the involvement of CeA to different forms of aversive transfer.

The results from this study replicate previously reported transfer effects of comparable strength with both shock-paired and klaxon-paired stimuli using a within-subjects design (Campese et al., 2017b). Both shock-paired and klaxon-paired cues generated motivation to comparably augment footshock avoidance behavior. While shock-paired stimuli are associated with sensory features of footshock, so is shock-avoidance behavior. Thus, facilitation of shock avoidance by a shock-paired cue likely involves sensory-specific properties of the shock outcome. This cannot be the case for a klaxon-paired cue, which can only augment USAA through general processes. Using KORD to inhibit neurons in CeA impaired transfer to both klaxon-paired and shock-paired stimuli; PIT was intact following vehicle treatment, but chemogenetic inhibition of CeA via KORDs attenuated the transfer effect.

In contrast, different forms of appetitive motivation have been found dependent upon parallel circuits in the amygdala involving CeA and Basolateral amygdala (BLA). According to studies in appetitive PIT, CeA is more sensitive to general motivation, while BLA regulates behavior based on sensory-specificity (Corbit and Balleine, 2005). However, mixed results have been found when testing conditions are similar to those used in the current study, and only include a single instrumental response. In this case, behavior is heavily dependent upon CeA (Holland and Gallagher, 2003). Alternatively, the difference may have to do with motivational modality. More work would be needed to directly address this discrepancy.

The manipulation of CeA with KORDs in this study did not produce freezing effects that could interfere with the expression of PIT and, therefore, provides strong evidence that CeA is important for generating modulatory effects of the CS on USAA, regardless of the signaled outcome. It should be acknowledged that while more specific than electrolytic lesions used in previous work, KORD expression was often found to extend beyond CeA into the Basal amygdala (BA) and dorsal medial to CeA in the current study. However, because prior studies have shown that BA lesions do not impair aversive PIT, the possibility that this could influence the effects of KORD is low. Since CeA inactivation reliably impaired PIT, BA activation was likely incidental. There is insufficient data to speculate as to how the dorsal medial spread of KORD may impact aversive PIT, but data showing contributions of the extended amygdala to fear conditioning suggest more work is needed to determine possible roles for these regions in avoidance and related phenomena (Ravinder et al., 2013).

In summary, these data provide strong evidence that CeA is important for the facilitative effect of aversive conditioned stimuli on active avoidance behavior. While avoidance itself is not dependent on CeA for acquisition or expression, the way acquired avoidance behavior may be integrated with prior experience appears to require changes to CS-processing in this region.

## Data Availability Statement

The raw data supporting the conclusions of this article will be made available by the authors, without undue reservation.

## Ethics Statement

The animal study was reviewed and approved by NYU University Animal Welfare Committee.

## Author Contributions

IK ran studies and did surgeries. CF processed tissue and quantified cell counts. MH processed tissue. SP, ET, and SC scored behavioral footage. VC designed the studies and wrote the manuscript. All authors contributed to the article and approved the submitted version.

## Conflict of Interest

The authors declare that the research was conducted in the absence of any commercial or financial relationships that could be construed as a potential conflict of interest.

## Publisher’s Note

All claims expressed in this article are solely those of the authors and do not necessarily represent those of their affiliated organizations, or those of the publisher, the editors and the reviewers. Any product that may be evaluated in this article, or claim that may be made by its manufacturer, is not guaranteed or endorsed by the publisher.

## References

[B1] Bolles R. C. Popp R. J. Jr. (1964). Parameters affecting the acquisition of Sidman avoidance. J. Exp. Anal. Behav. 7, 315–321. 10.1901/jeab.1964.7-315 14176280 PMC1404254

[B2] Campese V. D. Gonzaga R. Moscarello J. M. LeDoux J. E. (2015). Modulation of instrumental responding by a conditioned threat stimulus requires lateral and central amygdala. Front. Behav. Neurosci. 9:293. 10.3389/fnbeh.2015.00293 26578921 PMC4626560

[B5] Campese V. D. Soroeta J. M. Vazey E. M. Aston-Jones G. LeDoux J. E. Sears R. M.. (2017a). Noradrenergic regulation of central amygdala in aversive Pavlovian-to-instrumental transfer. eNeuro 4:ENEURO.0224–17.2017. 10.1523/ENEURO.0224-17.2017 29071299 PMC5654237

[B3] Campese V. D. Kim I. T. Rojas G. LeDoux J. E. (2017b). Pavlovian extinction and recovery effects in aversive Pavlovian to instrumental transfer. Front. Behav. Neurosci. 11:179. 10.3389/fnbeh.2017.00179 28993726 PMC5622165

[B4] Campese V. D. Kim J. Lázaro-Muñoz G. Pena L. LeDoux J. E. Cain C. K.. (2014). Lesions of lateral or central amygdala abolish aversive Pavlovian-to-instrumental transfer in rats. Front. Behav. Neurosci. 8:161. 10.3389/fnbeh.2014.00161 24847229 PMC4019882

[B6] Campese V. McCue M. Lázaro-Muñoz G. Ledoux J. E. Cain C. K. (2013). Development of an aversive Pavlovian-to-instrumental transfer task in rat. Front. Behav. Neurosci. 7:176. 10.3389/fnbeh.2013.00176 24324417 PMC3840425

[B7] Cardinal R. N. Parkinson J. A. Hall J. Everitt B. J. (2002). Emotion and motivation: the role of the amygdala, ventral striatum and prefrontal cortex. Neurosci. Biobehav. Rev. 26, 321–352. 10.1016/s0149-7634(02)00007-6 12034134

[B8] Cartoni E. Balleine B. Baldassarre G. (2016). Appetitive Pavlovian-instrumental transfer: a review. Neurosci. Biobehav. Rev. 71, 829–848. 10.1016/j.neubiorev.2016.09.020 27693227

[B9] Corbit L. H. Balleine B. W. (2005). Double dissociation of basolateral and central amygdala lesions on the general and outcome-specific forms of Pavlovian-instrumental transfer. J. Neurosci. 25, 962–970. 10.1523/JNEUROSCI.4507-04.2005 15673677 PMC6725628

[B10] Elrich J. C. Bush D. E. A. LeDoux J. E. (2012). The role of the lateral amygdala in the retrieval and maintenance of fear-memories formed by repeated probabilistic reinforcement. Front. Behav. Neurosci. 6:16. 10.3389/fnbeh.2012.00016 22514524 PMC3322351

[B11] Fadok J. P. Markovic M. Tovote P. Lüthi A. (2018). New perspectives on central amygdala function. Curr. Opin. Neurobiol. 49, 141–147. 10.1016/j.conb.2018.02.009 29522976

[B12] Fernando A. B. Urcelay G. P. Mar A. C. Dickinson A. Robbins T. W. (2014). Safety signals as instrumental reinforcers during free-operant avoidance. Learn. Mem. 21, 488–497. 10.1101/lm.034603.114 25135197 PMC4138357

[B13] Herry C. Johansen J. P. (2014). Encoding of fear learning and memory in distributed neuronal circuits. Nat. Neurosci. 17, 1644–1654. 10.1038/nn.3869 25413091

[B14] Holland P. C. Gallagher M. (2003). Double dissociation of the effects of lesions of basolateral and central amygdala on conditioned stimulus-potentiated feeding and Pavlovian-instrumental transfer. Eur. J. Neurosci. 17, 1680–1694. 10.1046/j.1460-9568.2003.02585.x 12752386

[B15] Johansen J. P. Cain C. K. Ostroff L. E. LeDoux J. E. (2011). Molecular mechanisms of fear learning and memory. Cell 147, 509–524. 10.1016/j.cell.2011.10.009 22036561 PMC3215943

[B16] Killcross S. Robbins T. W. Everitt B. J. (1997). Different types of fear-conditioned behaviour mediated by separate nuclei within amygdala. Nature 388, 377–380. 10.1038/41097 9237754

[B17] Lázaro-Muñoz G. LeDoux J. E. Cain C. K. (2010). Sidman instrumental avoidance initially depends on lateral and basal amygdala and is constrained by central amygdala-mediated Pavlovian processes. Biol. Psychiatry 67, 1120–1127. 10.1016/j.biopsych.2009.12.002 20110085 PMC3085029

[B18] LeDoux J. E. (2000). Emotion circuits in the brain. Annu. Rev. Neurosci. 23, 155–184. 10.1146/annurev.neuro.23.1.155 10845062

[B19] LeDoux J. E. Cicchetti P. Xagoraris A. Romanski L. M. (1990). The lateral amygdaloid nucleus: Sensory interface of the amygdala in fear conditioning. J. Neurosci. 10, 1062–1069. 10.1523/JNEUROSCI.10-04-01062.1990 2329367 PMC6570227

[B20] LeDoux J. E. Iwata J. Cicchetti P. Reis D. J. (1988). Different projections of the central amygdaloid nucleus mediate autonomic and behavioral correlates of conditioned fear. J. Neurosci. 8, 2517–2529. 10.1523/JNEUROSCI.08-07-02517.1988 2854842 PMC6569498

[B21] Lee J. L. Dickinson A. Everitt B. J. (2005). Conditioned suppression and freezing as measures of aversive Pavlovian conditioning: Effects of discrete amygdala lesions and overtraining. Behav. Brain Res. 159, 221–233. 10.1016/j.bbr.2004.11.003 15817185

[B100] Marchant N. J. Whitaker L. R. Bossert J. M. Harvey B. K. Hope B. T. Kaganovsky K.. (2016). Behavioral and physiological effects of a novel kappa-opioid receptor-based DREADD in rats. Neuropsychopharmacology 41, 402–409. 10.1038/npp.2015.149 26019014 PMC5130116

[B22] Martinez R. C. Gupta N. Lázaro-Muñoz G. Sears R. M. Kim S. Moscarello J. M.. (2013). Active vs. reactive threat responding is associated with differential c-Fos expression in specific regions of amygdala and prefrontal cortex. Learn. Mem. 20, 446–452. 10.1101/lm.031047.113 23869027 PMC3718200

[B23] Overmier J. B. Brackbill R. M. (1977). On the independence of stimulus evocation of fear and fear evocation of responses. Behav. Res. Ther. 15, 51–56. 10.1016/0005-7967(77)90087-0 836260

[B24] Overmier J. B. Payne R. J. (1971). Facilitation of instrumental avoidance learning by prior appetitive Pavlovian conditioning to the cue. Acta Neurobiol. Exp. Wars 31, 341–349. 5140158

[B25] Patterson J. Overmier J. B. (1981). A transfer of control test for contextual associations. Anim. Learn. Behav. 9, 316–321. 10.3758/BF03197837

[B26] Paxinos G. Watson C. (2005). The Rat Brain In Stereotaxic Coordinates, 6th Edn. New York, NY: Academic Press.

[B27] Ravinder S. Burghardt N. S. Brodsky R. Bauer E. P. Chattarji S. (2013). A role for the extended amygdala in the fear-enhancing effects of acute selective serotonin reuptake inhibitor treatment. Transl. Psychiatry 3:e209. 10.1038/tp.2012.137 23321806 PMC3566718

[B28] Rescorla R. A. (1968). Pavlovian conditioned fear in Sidman avoidance learning. J. Comp. Physiol. Psychol. 65, 55–60. 10.1037/h0025412 5648465

[B29] Rescorla R. A. Lolordo V. M. (1965). Inhibition of avoidance behavior. J. Comp. Physiol. Psychol. 59, 406–412. 10.1037/h0022060 14313781

[B30] Rogan M. T. LeDoux J. E. (1996). Emotion: systems, cells, synaptic plasticity. Cell 85, 469–475. 10.1016/s0092-8674(00)81247-7 8653782

[B31] Rosenkranz J. A. Grace A. A. (2002). Dopamine-mediated modulation of odour-evoked amygdala potentials during Pavlovian conditioning. Nature 417, 282–287. 10.1038/417282a 12015602

[B32] Sidman M. (1953a). Avoidance conditioning with brief shock and no exteroceptive warning signal. Science 118, 157–158. 10.1126/science.118.3058.157 13076224

[B33] Sidman M. (1953b). Two temporal parameters of the maintenance of avoidance behavior by the white rat. J. Comp. Physiol. Psychol. 46, 253–261. 10.1037/h0060730 13096610

[B101] Vardy E. Robinson J. E. Li C. Olsen R. H. J. DiBerto J. F. Giguere P. M.. (2015). A new DREADD facilitates the multiplexed chemogenetic interrogation of behavior. Neuron 86, 936–946. 10.1016/j.neuron.2015.03.065 25937170 PMC4441592

[B34] Weisman R. G. Litner J. S. (1969). Positive conditioned reinforcement of Sidman avoidance behavior in rats. J. Comp. Physiol. Psychol. 68, 597–603. 10.1037/h0027682

